# Astrocytes in Neurofluid Drainage and Brain Disorders: Mechanisms and Therapeutic Implications

**DOI:** 10.2174/011570159X406243251030105102

**Published:** 2026-01-05

**Authors:** Wenzhi Wang, Yichen Tsai, Yaojian Ding, Qinhan Yao, Jingru Tian, Yanrong Sun, Lihua Qin, Junhao Yan

**Affiliations:** 1 Department of Anatomy, Histology and Embryology, School of Basic Medical Sciences, Peking University Health Science Center, Beijing, 100191, China;; 2 Beijing Key Lab of Magnetic Resonance Imaging Technology, Peking University Third Hospital, Beijing, 100191 China

**Keywords:** Astrocyte, glymphatic system, intramural peri-arterial drainage, neurofluid, aquaporin 4, stroke, Alzheimer's disease

## Abstract

Over the past decade, increasing attention has been paid to neurofluid (NF) drainage in the brain, particularly to the glymphatic system and intramural periarterial drainage (IPAD) pathway, which are responsible for substance transport in the brain and are highly dependent on astrocyte function. The dysfunction of these drainage pathways can lead to the accumulation of toxic substances and fluids, and contribute to various brain diseases, such as stroke and Alzheimer’s disease. Since astrocytes in the brain closely connect to the microvascular system with their endfeet, in this work, the roles of astrocytes in regulating the glymphatic system and IPAD pathway and their dysfunction in neurodegenerative diseases have been comprehensively reviewed. Additionally, the effects of aquaporin 4, a water channel protein located on astrocytic endfeet, on these two pathways are explored. Furthermore, the possible therapeutic strategies for brain diseases targeting the NF drainage systems have also been proposed and thoroughly discussed.

## 1 INTRODUCTION

 In recent years, there has been a surge in exploring the function of astrocytes in neurofluid (NF; also described as interstitial fluid) drainage in the brain, which highlights their indispensable roles in the central nervous system (CNS) homeostasis [1, 2]. Previous studies have demonstrated that the dysfunction of NF drainage pathways results in compromised clearance of cerebral waste and aberrant proteins, which are notably observed in some neurodegenerative diseases, *e.g*., Alzheimer's disease [3, 4]. In fact, some distinct modes of NF outflow from the brain have been proposed, such as the glymphatic system [5] and the intramural periarterial drainage (IPAD) pathway [6]. It is believed that the functional integrity of NF drainage pathways is intricately regulated by astrocytes.

Astrocytic endfeet envelop brain microvessels, regulate ion homeostasis, support neurovascular signaling, and contribute to the formation of the blood-brain barrier (BBB) in coordination with endothelial cells and pericytes. The capillary-astrocyte complex is recognized as a significant contributor to NF production [3, 7]. Furthermore, astrocytes play a crucial role in substance transport by establishing selective permeability and maintaining BBB integrity. Perivascular astrocytes prominently express aquaporin-4 (AQP4) on their endfeet, facilitating water exchange between the vasculature and brain parenchyma [8]. Although much attention has been paid to astrocytic involvement in the glymphatic system, a comprehensive understanding of the relationship between astrocyte injury and NF drainage disorder in the brain during neurodegenerative diseases remains to be clarified.

This review aims to synthesize current advances in understanding the roles of astrocytes in regulating NF drainage through the glymphatic and IPAD systems, with a particular focus on their molecular mediators, such as AQP4. We further discuss how astrocytic injury contributes to drainage pathway dysfunction in stroke and Alzheimer’s disease and explore potential therapeutic strategies that target astrocyte-mediated neurofluid clearance to restore brain homeostasis.

## 2 THE FUNCTION OF ASTROCYTES IN THE GLYMPHATIC SYSTEM

**Fig. (1):**
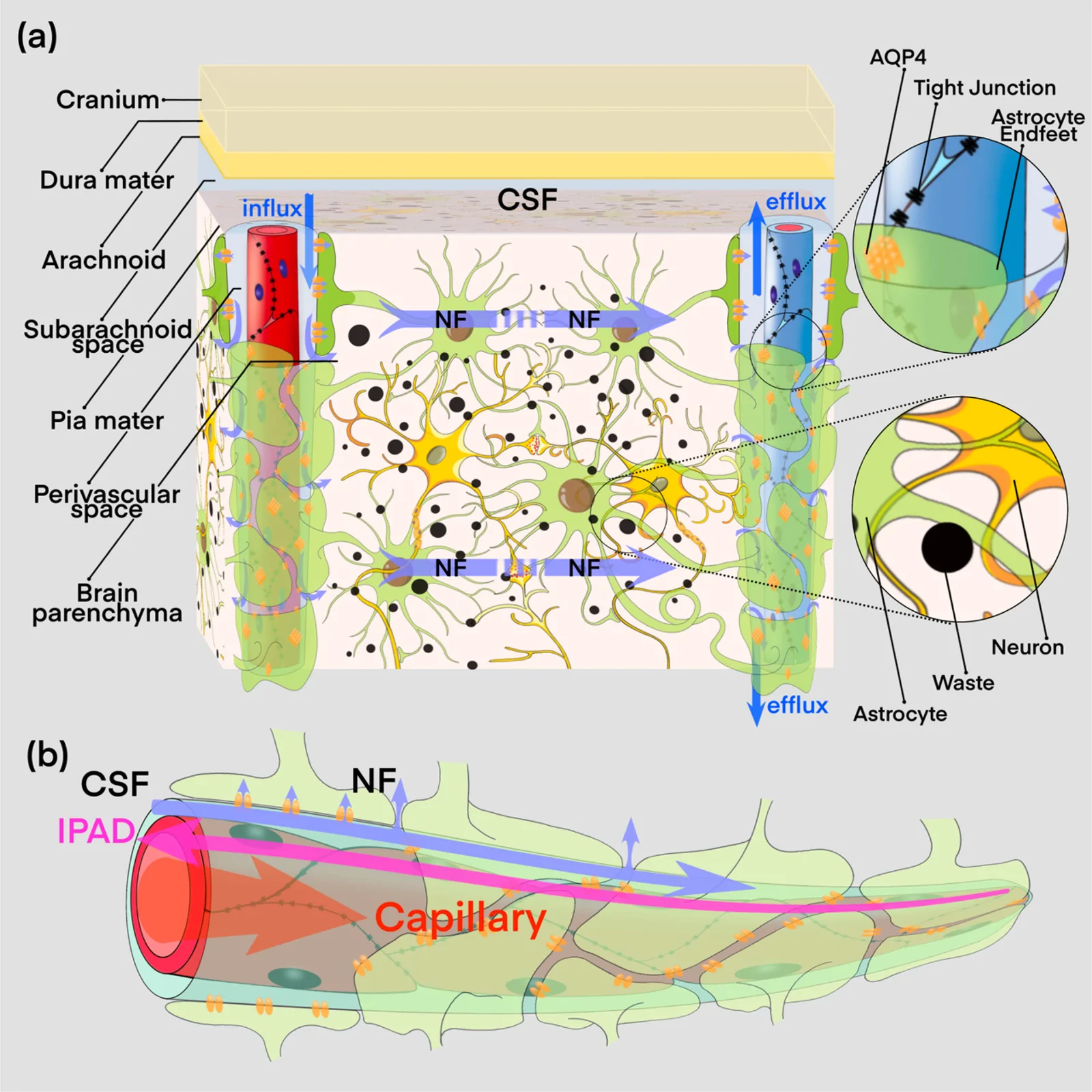
The anatomical structure and functional patterns of the glymphatic system and IPAD pathway. (**a**) The glymphatic pathway. CSF flows into the perivascular space (PVS), driven by smooth muscle cell (SMC) contraction, and mixes with neurofluid (NF), which is cleared along the PVS around the veins. All this process is facilitated by aquaporins (AQP4) located on astrocyte endfeet membranes. (**b**) The IPAD drainage pathway. The IPAD drainage pathway operates within the basement membrane surrounding SMCs in perforating arteries. Metabolite waste or toxic factors can be cleared through the IPAD pathway.

The glymphatic system, first proposed by Iliff *et al*. (2012), challenged the traditional view that the central nervous system lacks a lymphatic drainage system. The authors demonstrated the movement of hydrophilic solutes from cerebrospinal fluid (CSF) into the brain parenchyma through the perivascular space (PVS), which is formed by astrocytes encircling small perforating arteries in the subarachnoid space (SAS). The PVS, also termed the Virchow-Robin space, is filled with CSF [5]. This finding was further confirmed by studies using two-photon and fluorescence microscopy, as well as magnetic resonance imaging (MRI) [9]. As the arteries penetrate the brain parenchyma, their diameters gradually decrease and eventually merge with the vascular basement membrane, which contains another clearance system, the IPAD pathway (Fig. **1**) [10, 11].

 CSF is primarily produced in the lateral ventricles and flows into the SAS *via* the lateral and median apertures in the fourth ventricle. CSF enters the brain parenchyma *via* the PVS surrounding the cerebral arteries, mixes with interstitial fluid, and is subsequently cleared into the SAS along the PVS surrounding veins, a process regulated by polarized AQP4 on astrocytic endfeet [12, 13]. Thus, by regulating the PVS structure and AQP4 expression level or distribution, astrocytes facilitate the efficient drainage of NF within the brain. This process involves not only metabolic waste clearance but also the transportation of water-soluble molecules, *e.g*., glucose, and some lipid-soluble molecules [14, 15].

However, the driving force of CSF or NF movement in the glymphatic system remains under debate, with possible contributing factors including arterial pulsation, respiration, body position, and others [16]. Through modulating AQP4 and transforming some subtypes, astrocytes are also believed to closely regulate NF flow in the brain. Therefore, astrocytic injury often leads to NF drainage dysfunction and the development of some neurodegenerative diseases [17].

### 2.1 Aquaporin-4 Polarization and Astrocyte Subtypes in Neurofluid Regulation

Astrocytes are integral to NF homeostasis, particularly through their structural interactions with cerebral microvasculature and expression of specific ion and water channels. Among these, AQP4, a bidirectional water channel predominantly expressed on astrocytic endfeet, plays a central role in NF dynamics. AQP4 is composed of six bilayer-spanning domains and five connecting loops (A-E), with loops B and E containing conserved Asn-Pro-Ala (NPA) motifs forming a narrow aqueous pore (~2.8 Å). An arginine residue within the pore restricts protonated water and cation passage [18, 19].

Polarized localization of AQP4 on astrocyte endfeet adjacent to the pia mater and ependymal lining facilitates water exchange between CSF and interstitial fluid (ISF), supporting both CSF absorption and glymphatic function [16]. This polarization reduces hydraulic resistance, aiding water movement and NF flow, while also maintaining potassium buffering, glutamate homeostasis, and synaptic plasticity [20, 21]. NF movement is driven by a hydrostatic gradient across the extracellular space (ECS), which can be influenced by ion flux through astrocytes. Notably, AQP4 polarization is crucial to these processes [13].

Astrocytes also express other functional channels, such as the inward rectifier potassium channel Kir4.1, mediating potassium uptake and redistribution. This process helps modulate ECS volume, thereby influencing glymphatic transport efficiency [20, 22].

Dysfunction of AQP4 can lead to pathologies like brain edema [23]. In animal models, AQP4 deficiency reduces CSF influx and decreases NF clearance by up to 70% [5]. Enhancing AQP4 polarization, for instance *via* Fingolimod, improves glymphatic function, whereas agents like TGN-20 diminish polarization and impair drainage [24]. AQP4 also supports the circadian rhythm of CSF distribution. During sleep or anesthesia, PVS expansion and upregulated AQP4 expression enhance waste clearance, correlating with lower concentrations of amyloid-β and tau proteins [25, 26]. Conversely, single nucleotide polymorphisms (SNPs) in the AQP4 gene (*e.g*., rs72878776) are linked to poor sleep quality and increased risk of neurodegenerative disease [26-29]. PVS enlargement and white matter hyperintensities in Alzheimer's disease (AD) reflect fluid accumulation, with AQP4 expression higher in gray matter than white matter [27].

Crucially, AQP4 polarization is lost under pathological conditions, such as AD and stroke, leading to impaired drainage. In Parkinson’s disease, AQP4 abnormally accumulates near α-synuclein-positive neurons, further disrupting its distribution [13, 28, 29].

However, the role of AQP4 remains debated. Some studies report no significant impact of AQP4 knockout on tracer distribution in CSF or striatal clearance [30], though these findings are confounded by anesthesia protocols known to suppress glymphatic activity [31]. Experimental variables such as animal strain, age, and pharmacologic agents can greatly influence outcomes [12].

The depolarization of AQP4 is closely linked to astrocyte reactivity and subtype transitions. Astrocytes themselves are highly heterogeneous and capable of rapid structural changes in response to environmental stimuli. Their protrusions exhibit localized calcium signaling, and their morphological adaptations can persist from seconds to days [26]. Under pathological stress, astrocytes become reactive, transitioning into distinct phenotypes. A1 astrocytes (neurotoxic) dominate in neurodegeneration, impairing synaptic function, whereas A2 astrocytes (neuroprotective) emerge after ischemic injury and promote neuronal survival [22, 32-34]. These subtype shifts critically influence AQP4 polarization and glymphatic function [35, 36].

Neuroinflammation induces reactive astrocytes to release matrix metalloproteinases (MMPs), disrupting AQP4 expression and compromising drainage efficiency [37]. The resulting imbalance between pro- and anti-inflammatory signals promotes further astrocyte activation and contributes to NF stagnation.

Therefore, transitions between astrocyte subtypes underlie many of the pathological changes in drainage dynamics, weakening driving forces and exacerbating waste accumulation in the CNS. A comprehensive understanding of astrocyte changes in response to diverse stimuli holds promise for identifying potential risk factors in clinical settings.

In summary, astrocytes are central to neurofluid clearance through both their structural plasticity and regulation of AQP4. Future studies should address the interaction between astrocyte subtype switching, AQP4 regulation, and glymphatic dynamics, while accounting for confounding experimental variables such as anesthetic agents, animal strains, and age.

## 3 ASTROCYTES IN THE IPAD SYSTEM

The IPAD system was initially proposed by Carare’s team in 2008 [6, 38], and is composed of the basement membrane of cerebral arteries and arterioles, facilitating the directional transport of solutes rather than cells [6, 38]. Like an elastic porous medium, the IPAD system is filled with NF driven by contraction of vascular smooth muscle and can clear metabolic waste opposite to the direction of blood flow [39]. The IPAD plays a crucial role in metabolite clearance from the brain. Studies show that fluorescent β-amyloid injected into the SAS co-localizes with IPAD components during the clearance process, suggesting its involvement in clearing β-amyloid and preventing cerebral amyloidosis [40].

Initially, cerebral arterial pulsation was proposed as the primary driving force of intramural periarterial drainage. In 2013, Iliff and colleagues observed alterations in the exchange rate of perivascular CSF and NF by internal carotid artery ligation or phenylephrine administration, suggesting arterial pulsation might serve as the driving force of IPAD [16]. However, Diem's team proposed that arterial pulsation alone might be insufficient to drive IPAD [38]. They proposed a model suggesting that the rhythmic movement driven by astrocytes was propagated from the smooth muscle cells to the basement membrane, thereby indirectly driving solute efflux opposite to the direction of blood flow [41]. Aldea *et al*. proposed that smooth muscle contraction, instead of arterial pulsation, drives the IPAD system. Their model better explains the experimental results and challenged the pulsation hypothesis, suggesting that smooth muscle rhythmic pressure facilitates solute transport in the IPAD system [39].

 In the IPAD system, astrocytes play a pivotal role by regulating smooth muscle contraction, which consequently controls the driving force of the IPAD pathway. Additionally, astrocytes secrete basement membrane components that are crucial for IPAD formation [42]. These roles underscore astrocytes' significance in facilitating the drainage function of IPAD in the brain.

**Fig. (2):**
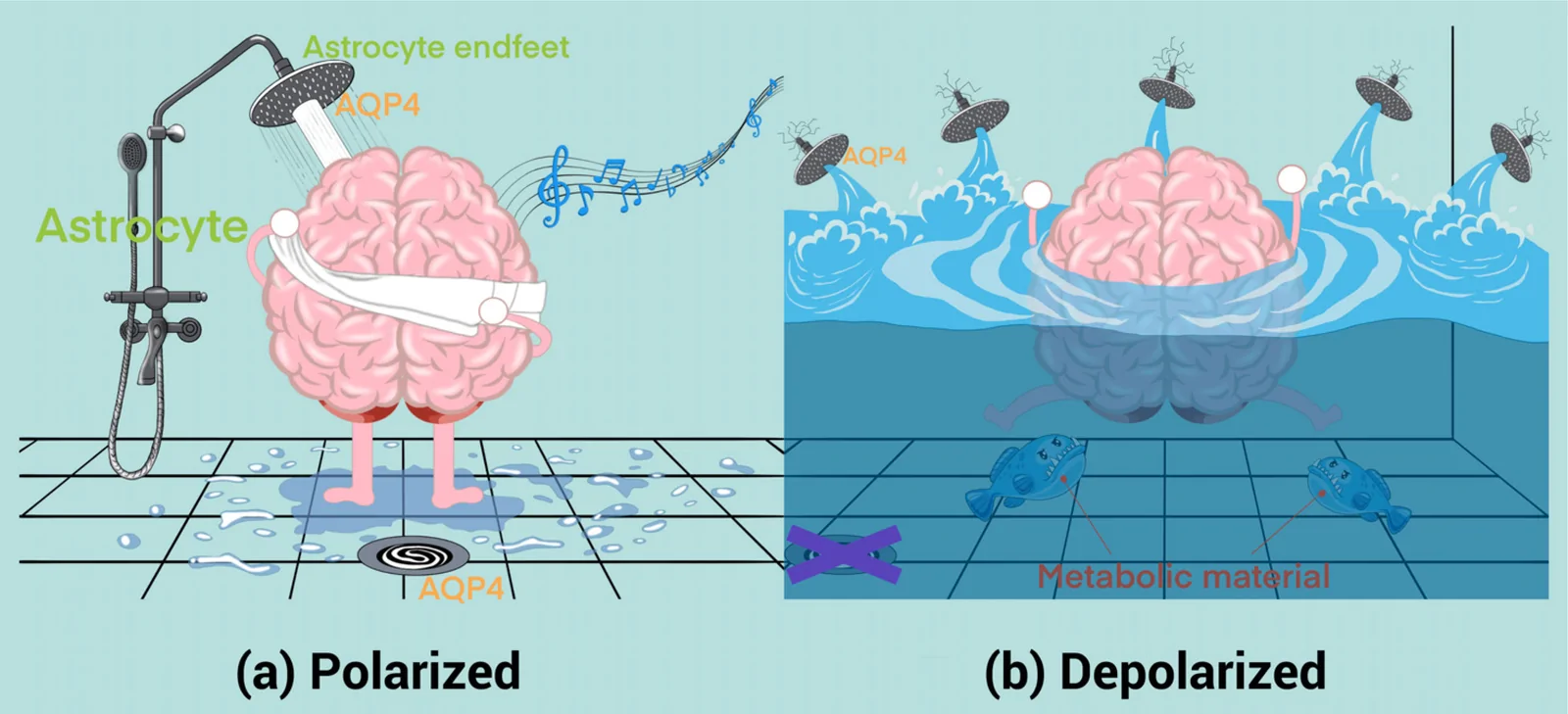
The influence of AQP4 polarization on brain function. (**a**) Highly polarized AQP4 plays a crucial role in CSF and NF exchange, thus maintaining brain health. In pathological conditions, such as stroke and AD, the expression level of AQP4 is significantly increased. (**b**) Additionally, AQP4 loses its polarization in reactive astrocytes. It is no longer localized around blood vessels, leading to dysfunction of the NF drainage pathway and consequent accumulation of excessive water and metabolic substances.

The neurovascular units (NVUs) comprise penetrating brain arteries, PVS, astrocytes, and surrounding neurons [43]. Besides serving as the physical barrier between arterioles and brain tissue, the NVUs also facilitate substance exchange. Studies indicate that astrocytes regulate nutrient transport by modulating tight junction density within NVUs [44] (Fig. **2**).

Within the NVU, astrocytes engage in bidirectional signaling between arterioles and neurons. Interestingly, astrocytes around PVS in the brainstem respond to cerebral hypoperfusion by activating neurons *via* calcium signaling pathways, thereby regulating heart rate and blood pressure to compensate for inadequate perfusion [45]. Astrocytes mediate neurovascular coupling by responding to hypoperfusion-induced alterations in perfusion and metabolic status.

Neurovascular coupling, also known as functional hyperemia, occurs in metabolically active brain regions undergoing vasodilation to increase blood supply. This process, observed using cerebral blood flow imaging, plays multiple roles, including enhancing perfusion to active brain areas, modulating brain temperature, and promoting CSF circulation, potentially aiding metabolite clearance [46, 47].

Despite growing interest in intramural IPAD as a key mechanism for cerebral waste clearance, several experimental limitations continue to constrain progress in the field. The fundamental limitation is the lack of direct imaging techniques capable of capturing real-time IPAD dynamics with the necessary spatial resolution. Most current evidence derives from tracer studies and static histological assessments, which provide indirect or snapshot views of solute movement along basement membranes [40], rather than continuous visualization of fluid flow. Although computational and mathematical models have proposed mechanisms such as vasomotion and functional hyperemia to drive IPAD, these remain largely theoretical due to limited *in vivo* validation [38, 39]. Even high-resolution methods like two-photon microscopy or advanced tracer analyses fail to reliably distinguish between periarterial and intramural compartments. Recent efforts using confocal microscopy and machine learning to trace therapeutic antibody clearance *via* IPAD in metastatic brain lesions illustrate the route’s importance but highlight continued reliance on post-mortem or fixed-tissue approaches [48]. Similarly, *in vitro* models of aging vasculature reveal altered basement membrane composition in response to hypercapnia and amyloid-β but cannot replicate the biomechanical or dynamic aspects of IPAD *in vivo* [49]. Compounding these challenges, blast injury models show early IPAD disruption *via* plasma leakage and basal lamina remodeling prior to overt inflammation, yet without technologies such as live electron microscopy, this pathology cannot be directly visualized or temporally resolved [50]. Together, these limitations underscore a critical need for advanced *in vivo* imaging techniques and experimental models to move from descriptive anatomy to a mechanistic understanding of IPAD.

**Fig. (3):**
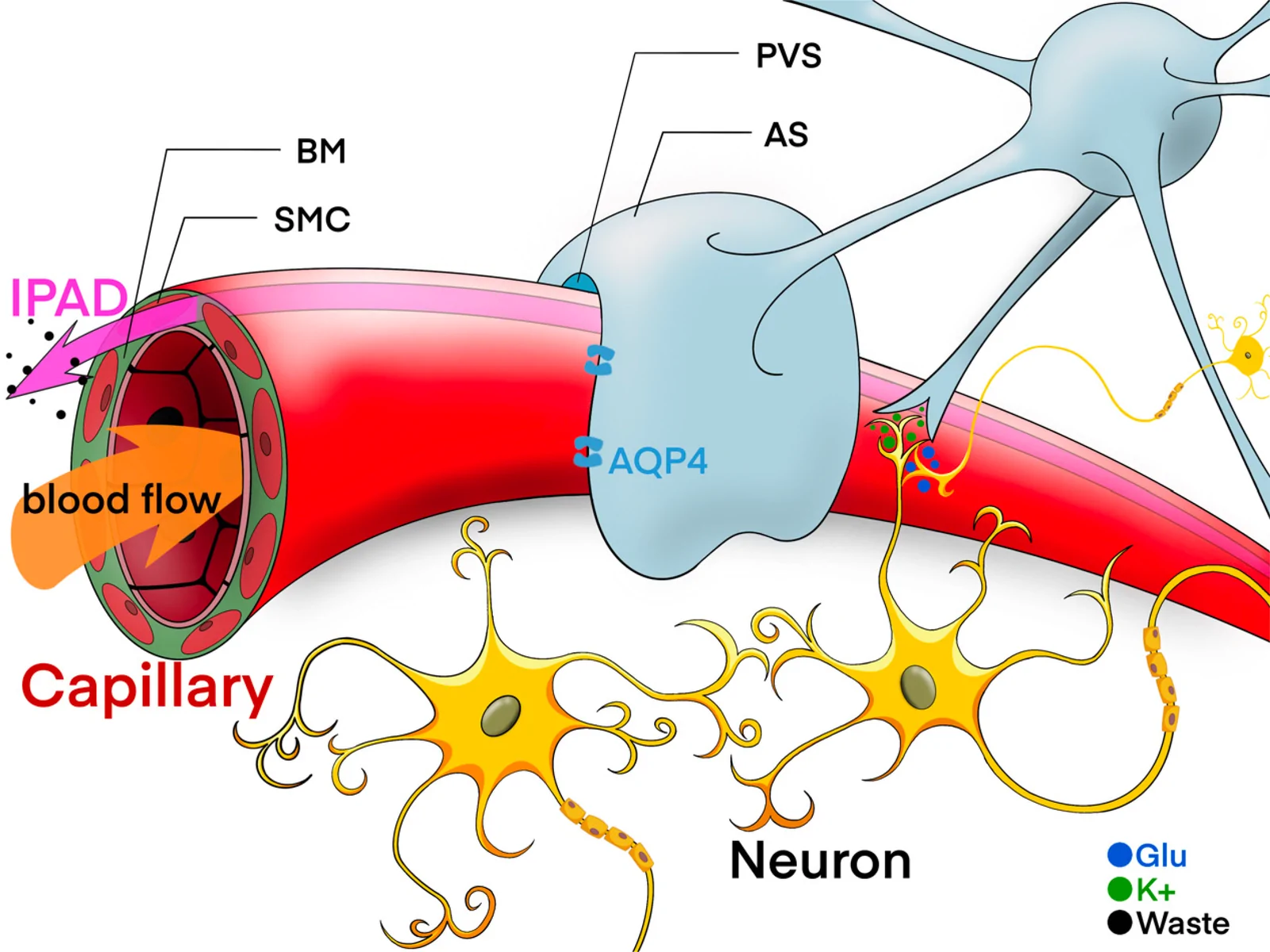
The roles of NVU in NF drainage. NVU is conducive to the neurovascular couples. Potassium ions (K^+^) in neurons participate in the generation of action potentials. This causes an increase in intracellular calcium ions in astrocytes, which then release vasodilators to relax arteriolar smooth muscle cells (SMCs) and raise regional blood flow. In this scenario, astrocytes can modulate the rhythmic movements of SMCs, thereby increasing the influx-driven force of CSF into the brain parenchyma and enhancing the NF outflux efficiency within the basement membrane in the IPAD pathway. BM: basement membrane; Glu: Glutamate.

Future research should focus on confirming the molecular mechanisms by which neurons can regulate the driving forces of IPAD and the glymphatic pathway, thereby determining whether some clinical interventions can enhance the driving force of NF drainage to preserve the neurophysiological microenvironment in the brain (Fig. **3**).

## 4 ROLES OF ASTROCYTES IN NF CLEARANCE DYSFUNCTION IN BRAIN DISORDERS

### 4.1 Roles of Astrocytes in NF Clearance Dysfunction Following Stroke

Stroke is classified into two main types: ischemic stroke and hemorrhagic stroke. Globally, ischemic strokes account for 60%-70% of all strokes and result from acute arterial occlusion. In contrast, hemorrhagic strokes make up approximately 20%-30% of strokes and occur due to the rupture of blood vessels, leading to bleeding in or around the brain [51].

Stroke significantly impairs NF clearance, regardless of the underlying cause (ischemia or hemorrhage). Both the glymphatic and IPAD pathways are compromised following vascular injury, resulting in the accumulation of toxic metabolites, brain swelling, and secondary neurodegeneration. In ischemic stroke, occlusion of cerebral arteries reduces oxygen and glucose supply, impairing interstitial solute clearance. In hemorrhagic stroke, such as subarachnoid hemorrhage or intraventricular hemorrhage (IVH), blood infiltration damages the vascular basement membrane, PVS, and endothelial glycocalyx, thereby enlarging PVS and hindering solute elimination through IPAD and glymphatic routes [27, 37, 38, 40].

Stroke leads to the accumulation of toxic substances in affected brain regions, which under normal conditions would be cleared *via* the glymphatic and IPAD pathways. However, damage to the PVS surrounding astrocytes, smooth muscle cell injury, and astrocyte activation themselves can compromise these clearance systems and worsen stroke outcomes [52].

A central molecular hallmark of drainage failure is AQP4 depolarization, which contributes to both cytotoxic and vasogenic edema. In the early stages of stroke, cellular energy failure and ionic imbalance lead to cytotoxic edema, characterized by sodium accumulation in astrocytes and subsequent water influx through AQP4 channels [27, 53]. This is followed by vasogenic edema, which results from upregulated AQP4 expression, loss of its perivascular polarity, and disruption of the BBB, allowing plasma proteins and interstitial fluid to accumulate in the brain parenchyma [27, 29, 54]. In both stroke types, AQP4 depolarization contributes to impaired neurofluid clearance. In parallel, impairment of astrocytes and associated damage to AQP4-coexpressed molecule, such as the excitatory amino acid transporter 2 (EAAT2), may lead to excessive extracellular glutamate accumulation, further disrupting the glutamine-glutamate-GABA cycle and contributing to excitotoxicity [55]. Additionally, loss of pericytes and BBB leakage in SAH result in compensatory AQP4 overexpression and glymphatic dysfunction [41].

Experimental evidence strongly supports the role of AQP4 in facilitating cytotoxic brain edema. In a foundational study [53], *Aqp4* knockout (*Aqp4*^-/-^) mice exhibited significantly reduced brain edema following both acute water intoxication and middle cerebral artery occlusion (MCAO). Specifically, brain water content in *Aqp4*^-/-^ mice was reduced by 2% compared to wild-type mice after water intoxication, and hemispheric swelling following MCAO was reduced by approximately 43%. These findings implicate AQP4 in astrocyte-mediated water influx during cytotoxic edema. Further supporting this role, Datta *et al*. demonstrated that endovascular administration of mesenchymal stem cells (MSCs) significantly downregulated AQP4 expression (43% reduction by Western blot) and decreased cerebral edema volume by 45% in a rat MCAO model [54]. Similarly, Gono *et al*. found that post-stroke treadmill exercise, which stimulated interleukin-1 receptor antagonist (IL-1RA) release, reduced AQP4 mRNA expression by approximately 46% and achieved a 1.7 percentage-point reduction in brain water content (81.2% → 79.5%; paired t-test), alongside a 39% reduction in edema volume as assessed by MRI [56]. Collectively, these studies provide compelling molecular and functional evidence that AQP4 upregulation promotes cytotoxic edema, while its suppression mitigates post-ischemic brain swelling.

 Astrocyte activation is a common pathological response in both stroke types. Post-stroke astrocytes undergo reactive astrogliosis, characterized pathologically by NLRP3 inflammasome activation, cytoskeletal remodeling (GFAP↑/ vimentin↓), and polarized redistribution of AQP4. A1-type reactive astrocytes exhibit AQP4 depolarization, contributing to glymphatic dysfunction and propagation of neuroinflammation [29]. These reactive changes often include swelling and protrusion of astrocytic endfeet, as seen in SAH, where they exacerbate vascular injury and disrupt neurovascular coupling through the release of pro-inflammatory cytokines [25, 39]. The interaction between AQP4 and the anchoring protein DP71 [57], which is reduced through ubiquitination after ischemia, further exacerbates AQP4 depolarization and reactive gliosis. Restoration of DP71 expression has been shown to enhance drainage and reduce brain edema [31]. These processes are coordinately regulated through multiple signaling pathways, including the Extracellular Signal-Regulated Kinase 1/2 (ERK1/2) cascade, which governs post-stroke neuroinflammation and AQP4 trafficking [20, 58].

Astrocytic gap junction communication, primarily mediated by connexins, may be altered under ischemic and hypoxic conditions. Some studies suggest that suppressing this intercellular coupling can protect astrocytes from hypoxia/reoxygenation injury [59]. This indicates that excessive gap junction activity may facilitate the diffusion of harmful signals, increasing cellular damage. In contrast, other studies report that, during early ischemia, gap junctions may support neuroprotection by enabling the transfer of beneficial molecules. For example, astrocytes may transfer mitochondria to injured neurons, providing metabolic support [60]. These findings suggest that the effects of gap junction communication are context-dependent. The mechanisms regulating this dual effect remain unclear and require further investigation.

The clinical implications of these findings are significant. AQP4 overexpression correlates with edema severity and may serve as a biomarker [32]. In animal models, inhibition of calmodulin (which regulates AQP4 membrane trafficking) or genetic knockout of AQP4 and Transient Receptor Potential Vanilloid 4 (TRPV4) reduces post-stroke edema and improves neurological outcomes [22, 28, 30]. Exercise-induced myokines such as IL-1RA also help mitigate AQP4 expression and edema in ischemia [33, 56]. In hemorrhagic stroke, while therapeutic clot lysis using recombinant tissue plasminogen activator (rt-PA) aids in clearing intraventricular blood, it also releases hemolytic products that impair astrocyte function and reduce drainage efficiency, sometimes worsening cerebral edema [43, 44]. Astrocytes also contribute to neuroprotection, for example by recycling damaged neuronal mitochondria and releasing synapse-protective cytokines [34, 35]. Region-specific drug effects on AQP4 have also been observed; the γ-secretase inhibitor DAPT (N- [N-(3,5-Difluorophenacetyl)-L-alanyl]-S-phenylglycine t-Butyl Ester) enhances AQP4 polarity in the striatum but not the cortex, emphasizing the importance of localized treatment strategies [36].

### 4.2 Roles of Astrocytes in NF clearance Dysfunction Following Alzheimer’s Disease

**Fig. (4):**
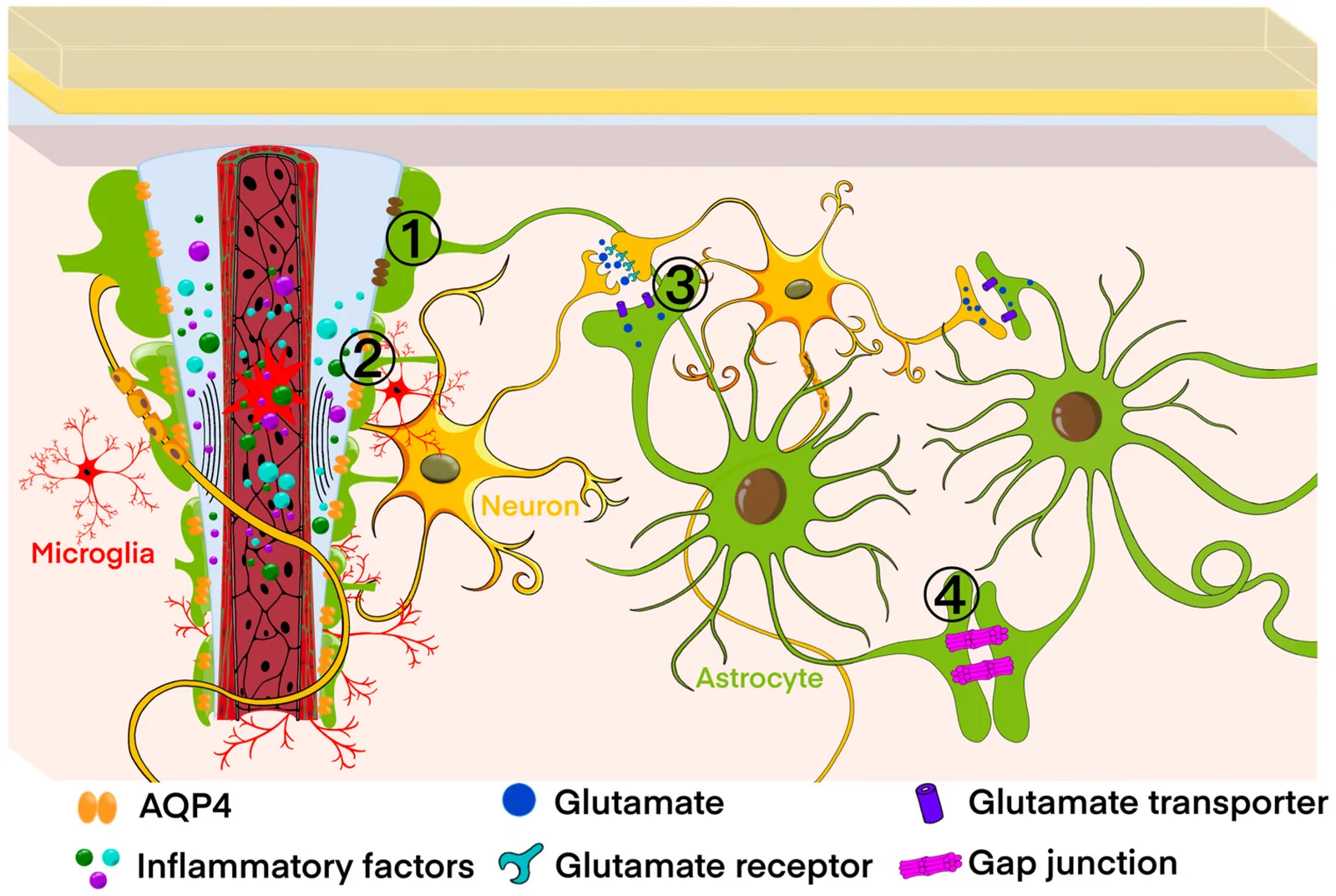
Roles of astrocytes in NF clearance dysfunction following stroke. **①** Stroke induces AQP4 depolarization and upregulation at astrocytic endfeet, disrupting glymphatic function and leading to brain edema and neurological deficits. **②** Astrocyte dysfunction after stroke compromises the integrity of the blood-brain barrier (BBB), exacerbating edema formation. **③** Dysfunction of the glutamate clearance system, mediated by impaired astrocytes, results in glutamate accumulation, leading to increased neuronal injury and edema. **④** Altered expression of astrocyte connexins may disrupt gap junction assembly and function, impairing intercellular communication.

 Alzheimer’s Disease (AD) manifests as progressive dementia, characterized by intracellular accumulation of beta-amyloid (Aβ) and tau protein, leading to the loss of episodic memory, language, visuospatial awareness, and executive function deterioration (Fig. **4**) [61-63].

Aβ and other parenchymal metabolites are partly cleared through the perivascular space by the glymphatic system. Alzheimer’s disease, as well as aging, with which AD strongly correlates, impair the function of glymphatic clearance by attenuating arterial pulsation, reducing CSF-ISF exchange, and diminishing lymphatic drainage, leading to the pathogenic accumulation of amyloid and tau [64]. The accumulation of pathogenic protein exacerbates neuroinflammation and causes further damage to the clearance system, promoting the progression of AD.

Research shows that AQP4 depolarization [65] and loss of localization on astrocyte endfeet [62, 66], as well as pathological enlargement of PVS and alterations in basal lamina proteins [17, 11], may be the underlying cause of pathogenic protein accumulation. Interestingly, postmortem tissue analysis shows that CSF AQP4 level significantly increases in the aging brain, while perivascular AQP4 localization decreases in AD patients compared with cognitively intact individuals and was negatively associated with age in the AD group [67]. The increased CSF AQP4 level and lost localization may be caused by neuroinflammation and A1 subtype reactive astrocytes [33], which requires further investigation.

Aquaporin changes in AD patients are not limited to AQP4 and astrocytes. Previous studies suggest reduced AQP expression in the choroid plexus of AD patients, which hinders CSF production, causing decreased pressure of CSF from the periarterial space to the brain parenchyma, further impairing glymphatic function [68]. Enlarged perivascular spaces, detectable on MRI, commonly occur in brain regions like the basal ganglia and white matter [4, 14, 17]. Sleep disturbances are common in AD patients and may exacerbate cognitive decline, as sleep enhances glymphatic function and Aβ clearance [69]. Conversely, AD pathophysiology may disrupt sleep patterns, suggesting a bidirectional relationship [70-72].

Studies have focused on glymphatic transport to identify new solutions to the disease. Sleep has been identified as a key factor in glymphatic function and CSF exchange, with a 25%-30% increase in amyloid clearance during sleep compared to wakefulness [72]. As a result, anesthetic agents including Ketamine-Xylazine have been used to enhance glymphatic function [73]. Research suggests multisensory 40 Hz stimulation can reduce the amyloid burden of 6-month-old 5XFAD mice *via* the glymphatic system, which is supported by follow-up experiments showing increased CSF tracer and ISF efflux rates in multisensory 40 Hz-treated mice [64]. Impaired glymphatic function in TGN020-treated or AQP4 knockout mice attenuated multisensory 40 Hz-mediated amyloid clearance, supporting the previously suggested function of glymphatic transport in metabolite clearance. Multisensory gamma stimulation improves glymphatic function by increasing arterial pulsation (the supposed major driving force of the glymphatic system) and upregulating genes contributing to polarization and localization of AQP4 [64].

Recent research has proposed cervical deep lymphatic-venous anastomosis (LVA) as a novel surgical strategy to enhance glymphatic-meningeal lymphatic clearance in Alzheimer’s disease (AD) [74]. The deep cervical lymph nodes serve as the terminal drainage route for cerebrospinal and interstitial fluid, linking glymphatic output to systemic lymphatic circulation. Impairment of these lymph nodes due to aging or chronic inflammation has been associated with CSF stagnation and pathological accumulation of amyloid-β and tau. Parallel preclinical research has demonstrated that LVA in mouse models enhances brain lymphatic clearance and reduces protein aggregation [75].

However, LVA remains a controversial intervention. Although a growing body of preclinical and clinical research supports the role of impaired glymphatic clearance in AD, no large-scale randomized clinical trials have formally evaluated LVA for AD treatment. Nevertheless, isolated case reports claiming symptomatic improvement have gained widespread attention on Chinese public online platforms [76], and the treatment has rapidly expanded to over 30 clinical centers across China. By the end of 2024, over 500 cases had been publicly reported, with hospital and media sources estimating that the actual number may exceed 1,000 [77]. This rapid clinical adoption without rigorous trial data has raised concern among some in the scientific and medical community, who caution that the causal relationship between LVA and cognitive improvement remains speculative [78]. The procedure’s invasive nature, combined with the lack of long-term safety and efficacy data, underscores the urgent need for controlled trials before LVA can be validated as a therapeutic approach in AD treatment.

## 5 DISCUSSION

In recent years, much attention has been paid to the glymphatic system and the IPAD pathway (Table **1**). This review has summarized the key roles of astrocytes in NF clearance. These include the polarized expression of AQP4, the distinct functions of astrocyte subtypes, and the release of bioactive molecules under various physiological and pathological conditions. The involvement of astrocytes in impaired NF drainage after stroke and AD has also been discussed.

However, contradictory findings in the literature complicate current understanding of astrocyte-regulated clearance mechanisms *via* the glymphatic and IPAD pathways.

First, while some studies (*e.g*., Iliff *et al*., 2012) emphasize the importance of AQP4 in promoting CSF-interstitial fluid exchange and solute clearance, others (*e.g*., Smith *et al*., 2017) report no significant effect of AQP4 deletion. These inconsistencies may reflect differences in experimental design, including anesthesia, pharmacological agents, animal strain, and age. This variation highlights the need for standardized protocols.

Second, the primary driving force of glymphatic flow remains debated. Arterial pulsation was initially proposed as the main mechanism. However, subsequent research suggests it may be insufficient. Alternative forces, such as smooth muscle contractions (Aldea *et al*., 2019) or astrocyte-related rhythmic fluctuations, may dominate under specific conditions. These findings indicate that glymphatic flow is likely governed by multiple mechanisms that vary with context.

Third, drugs intended to restore AQP4 polarity, such as Fingolimod and DAPT, have shown potential to improve glymphatic function. However, their effects are not uniform across brain regions. For example, DAPT increases AQP4 polarity in the striatum but not the cortex [36]. This raises questions about their overall applicability. In addition, the long-term safety and specificity of AQP4-targeted therapies remain uncertain. Unintended effects on other astrocytic or neurovascular functions may compromise outcomes.

A major challenge is the dual role of AQP4 in brain edema. AQP4 upregulation during the acute phase of stroke contributes to both cytotoxic and vasogenic edema, due to increased water transport and BBB disruption. In contrast, during the recovery phase, AQP4 aids in fluid clearance and edema resolution. This opposing function complicates drug development: AQP4 inhibition may reduce early edema but interfere with later recovery; enhancement of AQP4 may have the reverse effect.

Further complexity arises in the context of subarachnoid hemorrhage. While glymphatic and IPAD systems can help remove blood-derived waste, agents like recombinant tissue plasminogen activator (rt-PA) may impair astrocytic function and worsen edema. These effects create additional challenges for clinical intervention.

Altogether, these inconsistencies underline the complexity of astrocyte-mediated fluid regulation. Critical evaluation of AQP4-targeted strategies is needed. Future studies must account for factors such as timing, brain region, and disease context when developing therapeutic approaches.

This review also has limitations. First, the architecture of the glymphatic system, especially venous outflow routes, remains incompletely characterized. Lack of ultrastructural evidence, such as from electron microscopy, limits our anatomical understanding. Second, the clinical relevance of findings related to glymphatic and IPAD function is still poorly explored and requires more focused investigation.

To advance the field, we propose three main directions. First, the molecular mechanisms by which astrocytes regulate glymphatic and IPAD function should be examined using genetically modified animal models. Second, high-resolution imaging tools, including electron microscopy, should be employed to visualize perivascular and venous drainage structures. Third, translational studies should assess whether AQP4 expression can serve as a biomarker of neurodegenerative disease progression or guide targeted strategies to enhance waste clearance and support neurovascular stability.

## CONCLUSION

Recent studies have provided important insights into how astrocytes regulate neurofluid drainage through the glymphatic and IPAD systems. Central elements of these processes include the polarized localization of AQP4 at astrocytic endfeet and the integration of astrocyte activity within the neurovascular unit. These features support the movement and clearance of CSF and interstitial solutes. Under disease conditions, such as stroke and Alzheimer’s disease, changes in astrocyte subtypes and activation of inflammatory pathways further influence these clearance mechanisms.

Despite this progress, several questions remain unresolved. The physical forces that drive fluid movement are not fully understood. The fine structure of venous outflow pathways has not been clearly defined. Regional variation in astrocyte responses also complicates efforts to generalize findings. The dual function of AQP4, contributing to both edema formation and resolution, adds further complexity to drug development.

Future research should focus on combining high-resolution imaging methods, such as electron microscopy, with molecular biology techniques to better define drainage structures and signaling mechanisms. These efforts are essential for developing therapies that improve fluid clearance, protect the BBB, and reduce secondary injury in cerebrovascular and neurodegenerative diseases.

## Figures and Tables

**Table 1:** Summary of Glymphatic and IPAD Dysfunction in brain disorders.

**Category**	**Normal Conditions**	**Post-Stroke Conditions**	**Alzheimer’s Disease (AD) Conditions**	**References**
Drainage System	Functional glymphatic and IPAD pathways; Narrow PVS; Perivascular AQP4 polarization	Impairment of both pathways; PVS enlargement; AQP4 depolarization and mislocalization		[27, 29, 37, 38, 40, 41, 52, 58]
Astrocyte Physiology	Quiescent astrocytes; Support BBB and clearance; DP71 anchoring maintains AQP4 polarity	A1-type reactive astrocytes; Inflammatory cytokines; Loss of AQP4 polarity		[25, 29, 31, 33, 39, 52, 53, 59-62]
		Loss of DP71 promotes AQP4 depolarization	-	
Edema and BBB Integrity	Stable fluid homeostasis; Intact BBB prevents plasma leakage.	Cytotoxic edema (Na^+^/water influx); Vasogenic edema (plasma protein leakage); BBB breakdown	Enlarged PVS; Disrupted CSF production; Reduced clearance efficiency	[4, 14, 17, 27, 29, 41, 63]
Inflammatory & Signaling Pathways	Minimal basal inflammation; ERK1/2 inactive	ERK1/2 activation drives AQP4 mislocalization and gliosis	Chronic inflammation contributes	[20, 33, 54, 59, 62]
Clinical Relevance & Therapy	Efficient clearance; Homeostatic regulation of CSF-ISF exchange	AQP4 correlates with edema severity; Therapeutic targets include AQP4/TRPV4, calmodulin, IL-1RA, DP71	AQP4 as potential biomarker; 40 Hz stimulation, IL-1RA, LVA explored as interventions	[22, 28, 30-33, 36, 58, 62, 67-71]

## LIST OF ABBREVIATIONS

AD: Alzheimer's Disease
BBB: Blood-brain Barrier
CNS: Central Nervous System
CSF: Cerebrospinal Fluid
ECS: Extracellular Space
IPAD: Intramural Periarterial Drainage
LVA: Lymphatic-venous Anastomosis
MMPs: Matrix Metalloproteinases
MSCs: Mesenchymal Stem Cells
NF: Neurofluid
NVUs: Neurovascular Units
PVS: Perivascular Space
rt-PA: Recombinant Tissue Plasminogen Activator
SAS: Subarachnoid Space
SNPs: Single Nucleotide Polymorphisms
